# Predictive Modelling of Exam Outcomes Using Stress-Aware Learning from Wearable Biosignals

**DOI:** 10.3390/s25185628

**Published:** 2025-09-09

**Authors:** Sham Lalwani, Saideh Ferdowsi

**Affiliations:** School of Mathematics, Statistics and Actuarial Science, University of Essex, Colchester CO4 3SQ, UK; sl23849@essex.ac.uk

**Keywords:** academic performance prediction, physiological signals, machine learning, wearable sensors, stress detection

## Abstract

This study investigates the feasibility of using wearable technology and machine learning algorithms to predict academic performance based on physiological signals. It also examines the correlation between stress levels, reflected in the collected physiological data, and academic outcomes. To this aim, six key physiological signals, including skin conductance, heart rate, skin temperature, electrodermal activity, blood volume pulse, inter-beat interval, and accelerometer were recorded during three examination sessions using a wearable device. A novel pipeline, comprising data preprocessing and feature engineering, is proposed to prepare the collected data for training machine learning algorithms. We evaluated five machine learning models, including Random Forest, Support Vector Machine (SVM), eXtreme Gradient Boosting (XGBoost), Categorical Boosted (CatBoost), and Gradient-Boosting Machine (GBM), to predict the exam outcomes. The Synthetic Minority Oversampling Technique (SMOTE), followed by hyperparameter tuning and dimensionality reduction, are implemented to optimise model performance and address issues like class imbalance and overfitting. The results obtained by our study demonstrate that physiological signals can effectively predict stress and its impact on academic performance, offering potential for real-time monitoring systems that support student well-being and academic success.

## 1. Introduction

Over the past two decades, growing attention has been paid to the impact of psychological stress on academic performance. The relationship between stress and academic performance is nuanced; while moderate stress may enhance performance, excessive stress is often detrimental, as described by the Yerkes–Dodson law [1]. In educational contexts, stress commonly arises in two forms; acute stress, which occurs in response to short-term challenges such as examinations, and chronic stress, which develops over extended periods due to sustained academic or social pressures. Exam-related as a type of acute stress can impair cognitive function, cause restlessness, and reduce overall well-being, which is closely linked to academic underperformance [2]. Physiological stress responses, which reflect autonomic nervous system activity, provide valuable insights into how students cope during exams and can inform strategies to enhance both performance and well-being. Recent advances in wearable technologies now enable non-invasive, real-time monitoring of biomarkers such as heart rate (HR), heart rate variability (HRV), electrodermal activity (EDA), and skin temperature. Several studies have reported that wearable data can predict academic outcomes with accuracies of 70–80% [3,4,5], demonstrating the potential of physiological monitoring in educational settings.

In a recent study, HR and HRV were collected from 103 Japanese university students using smart rings to identify cyclical stress patterns across the academic year [6]. Both undergraduate and graduate students participated over a two-semester data collection period. Daytime (waking) HR, nighttime (sleep) HR, and nighttime (sleep) HRV were used as their chronic stress indicators. Sleep-related measures, including sleep duration, sleep stage percentages, sleep efficiency, and restless periods were also analyzed for additional insights. A linear regression model was then applied to examine the relationship between physiological and behavioral data and stress. Their findings highlighted the potential of wearable devices to detect collective changes in stress biomarkers within student cohorts.

Machine learning techniques have shown promise in predicting stress levels based on physiological data [7,8,9]. These techniques can classify different states of stress and their correlation with academic performance, thereby providing early indicators for students at risk of underperformance [10,11]. Despite progress, existing predictive models often struggle with signal complexity and individual variability [12]. Advanced algorithms and robust preprocessing techniques are needed to improve the reliability and generalizability of stress prediction systems. In this study, we investigate the potential of wearable sensors for predictive modelling of acute exam stress. We analyze physiological signals recorded during examination conditions using advanced machine learning techniques. We evaluate five models, including Random Forest (RF), Support Vector Machine (SVM), eXtreme Gradient Boosting (XGBoost), Categorical Boosting (CatBoost), and Gradient Boosting Machine (GBM), to interpret physiological data recorded during exams and correlate them with exam outcomes.

Abd-Alrazaq et al. [13] conducted a systematic review to evaluate the effectiveness of machine learning and wearable devices in detecting stress among students. Analyzing 19 studies (6 in meta-analysis), they reported a pooled accuracy of 85.6%, with sensitivity and specificity around 75%. Results highlight the promise of wearable AI for stress detection but emphasize the need for further refinement before stand alone application. Lisetti and Nasoz [14] studied the usage of non-invasive wearable computers to monitor galvanic skin response, heart rate, and temperature to recognize emotions. They used supervised learning methods including K-nearest neighbors (KNN), Deterministic Finite Automaton (DFA), and Marquardt Backpropagation (MBP) to classify emotions like melancholy, rage, fear, and amusement. Their analysis showed promising results in classifying the emotions based on the physiological measurements.

Amin et al. [15] conducted a lab-based stress detection study with 35 undergraduate students, using both research-grade (Biopac MP160, Polar H10) and consumer-grade (Empatica E4, Garmin Forerunner 55s) wearables to collect heart rate variability and electrodermal activity data. They used the support vector machine for classifications based solely on HRV, and Random Forest when combining HRV and EDA, using leave-one-subject-out (LOSO) cross-validation to assess robustness. Results showed that combining HRV with EDA significantly enhanced stress detection accuracy—Polar H10 and Garmin Forerunner 55s achieved AUROCs up to 0.954 and 0.961 respectively—highlighting the potential of consumer wearables for practical and scalable stress monitoring.

Li and Liu [16], developed a method using a deep neural network to predict stress based on physiological signals collected from the chest and wrist. In this study, six sensors were attached to subjects’ chests to collect electrocardiogram, electrodermal activity, electromyography, respiratory rate, and skin temperature. Additionally, a 3-axis accelerometer (ACC) sensor was attached to the subjects’ chests to measure 3-dimensional body movement information. Wrist-worn sensors collected blood volume pulse (BVP), EDA, skin temperature, and ACC. They employed a 1D Convolutional Neural Network (CNN) for chest data and a Multilayer Perceptron (MLP) for wrist data, enabling automatic feature learning from raw input. Their models achieved high accuracy in binary stress detection (i.e., stress and baseline) and multi-class (i.e., baseline, stressed, and amused) classification.

In another study, Hovsepian et al. [17] utilized wearable sensors to collect physiological signals including heart rate, breathing, and electrodermal activity for real-time stress detection in both lab and real-life settings. SVM was trained for stress classification, with preprocessing techniques addressing noise and physical activity interference. A total of 26 subjects participated in the lab study and 20 subjects participated in the real-life study setting. Their model achieved 98% recall for stress prediction using the lab data and 72% accuracy for the real-world condition.

The reviewed literature validates the integration of wearable sensing, robust preprocessing, and advanced machine learning techniques for high-fidelity stress prediction. While models show strong performance, challenges remain; particularly around individual variability, data quality, and context-awareness. This paper builds on existing insights by incorporating advanced preprocessing, feature engineering, and model tuning to enhance predictive accuracy and robustness. In this study, we used the physiological dataset collected by [10,18] to study the students’ cognitive performance in different exams. We also investigated the correlation of the exam street with students’ performances.

## 2. Material and Method

### 2.1. Data Collection

**Figure 1:**
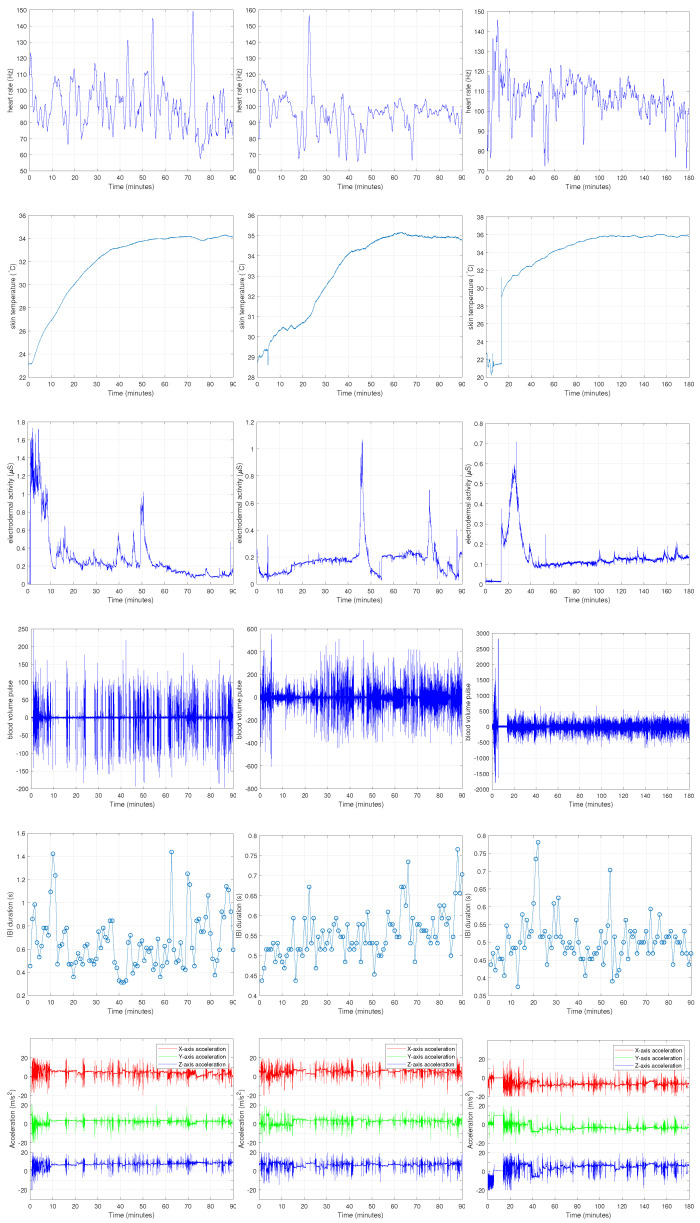
Six physiological signals that were collected from subject 2. The **left**, **middle**, and **right** columns correspond to midterm 1, midterm 2, and the final exam, respectively.

In this study, we applied our advanced data processing pipeline to the dataset collected by Amin et al. [19] using the Empatica E4 wearable device. This dataset is available publicly and can be downloaded from PhysioNet data archive [11,14]. Ten students, including two females and eight males, participated in data collection by wearing the device during three exam sessions, including midterm 1, midterm 2, and final exams. Participants were asked to read and sign an informed consent form prior to enrolling in the study. The E4 device was given to students five minutes earlier than the exams for data collection during the exams. Students wore the E4 device in their nondominant hand. The E4 wristband (produced by Empatica Inc., Cambridge, MA, USA) is a multi-sensor wearable device designed for real-time data acquisition in studies involving psychophysiology and behavioral monitoring. The length of the midterm 1 and midterm 2 exams was 1.5 h, and the length of the final exam was 3 h. Six physiological signals, including heart rate (HR), skin temperature (TEMP), electrodermal activity (EDA), blood volume pulse (BVP), inter-beat interval (IBI), and accelerometer (ACC) were recorded during the exams. In our study, physiological signals were collected at different sampling rates depending on the modality. Blood volume pulse was sampled at 64 Hz, electrodermal activity and skin temperature at 4 Hz, and accelerometer data at 32 Hz, while heart rate was derived at 1 Hz and inter-beat interval was stored as an event-based series. These rates were chosen to balance temporal resolution with efficient data acquisition for real-time monitoring. Figure 1 represents the physiological signal which were collected from subject 2. Signals collected during midterm 1 are shown in the left column, signals collected during midterm 2 are shown in the middle column, and signals collected during the final exam session are shown in the right column. The study protocol was reviewed and approved by the University of Houston Institutional Review Board (IRB). Since the main aim of this dataset was to explore the relationship between stress levels and exam performance, the academic scores were used to label the data. We addressed a binary classification problem in our analysis. To this aim, a threshold of the exam score has been used to assign labels of good and bad to the collected signals. The selected thresholds for midterm 1, midterm 2, and the final exam were 80, 100, and 200, respectively. The signals associated with scores above the selected thresholds were labelled as good and signals associated with scores below the selected thresholds labelled as bad. After labelling, six bad labels and four good labels were assigned to the data collected at each midterm. The labels for the final exam included five bad labels and five good labels. These labels were then used as the target variable for classification [10].

### 2.2. Preprocessing and Feature Engineering

Before applying the machine learning algorithms, the data underwent a series of preprocessing steps to ensure its suitability for model training and evaluation. Our preprocessing pipeline involved cleaning missing values and applying smoothing filters to reduce noise. A high pass filter with low cut off frequency of 0.5 Hz applied to blood volume pulse and accelerometer signals. Additionally, a low pass filter with cut off frequency 2 Hz is used to remove noise from EDA. The cut-off frequencies were selected through empirical tuning. Visual inspection indicated that applying this filter effectively removed low-frequency drifts in these signals, leading to more accurate feature extraction. Missing values in physiological data were handled by finding the mean of the available data points in the relevant columns and filling in the gaps. This strategy maintains data continuity while avoiding considerable bias caused by outliers or non-representative values. Then, statistical features were extracted from the physiological data. To this aim, first the physiological signals were divided into smaller segments using a one-minute window length and a 50% overlap between consecutive windows. A preliminary investigation was conducted by evaluating multiple window sizes, which indicated that the selected configuration is appropriate. Second, the statistical features including, mean, standard deviation, maximum, and minimum values for HR, TEMP, EDA, BVP, IBI, and ACC have been estimated. These features were computed for all signals collected during each exam session to quantify student stress responses in each condition. Data normalisation was also performed to scale the features uniformly. The main aim of normalisation was to ensure numerical stability and fair feature contribution. This involved scaling features to a common range or distribution so that no feature disproportionately influences the model due to higher magnitudes.

### 2.3. Model Training and Evaluation

We used five machine learning models, including Random Forest, Support Vector Machine, eXtreme Gradient Boosting, Categorical Boosting, and Gradient Boosting Machine, to investigate how the measured physiological signals can be used to predict academic performance. Each model was trained on the extracted features using 3-fold stratified cross-validation. It is important to note that the models were trained and evaluated independently for each examination session. In this study, the GridSearchCV technique was employed to perform exhaustive hyperparameter tuning for optimal performance. Additionally, two resampling methods, including Synthetic Minority Oversampling Technique (SMOTE) followed by ANOVA-based feature selection, were used for each model. The utilised resampling methods are statistical methods used to generate new data points in the dataset by randomly picking data points from the existing dataset. Resampling was incorporated within the fold to prevent data leakage. It helps in creating new synthetic samples for training machine learning models and in estimating the properties of a dataset when the dataset is unknown, difficult to estimate, or when the sample size of the dataset is small. Resampling was followed by dimensionality reduction using Principal Component Analysis (PCA) to enhance model interpretability and reduce overfitting.

Three approaches were used to train each machine learning model. These include (i) training and evaluation GridSearchCV, (ii) training and evaluation using GridSearchCV and resampling techniques, and (iii) training and evaluation using GridSearchCV, resampling and dimensionality reduction techniques. After training all the models using these approaches, the trained models were tested, and the obtained results were compared to identify the best data processing pipeline. Table 1, Table 2 and Table 3 summarize the optimal configurations for each strategy.

We implemented the data processing pipeline using Python 3.13.7 libraries including pandas, matplotlib, seaborn, and scikit-learn. Models’ performances were evaluated using Accuracy, Precision, Recall, and F1-score. Equations (1)–(4) represent the mathematical formulation of these indices.

(1) $$\mathrm{Accuracy}=\frac{TP+TN}{TP+TN+FP+FN}\times 100\%$$

(2) $$\mathrm{Precision}=\frac{TP}{TP+FP}\times 100\%$$

(3) $$\mathrm{Recall}=\frac{TP}{TP+FN}\times 100\%$$

(4) $$F1-\mathrm{score}=\frac{2\times \mathrm{Precision}\times \mathrm{Recall}}{\mathrm{Precision}+\mathrm{Recall}}$$

Here,

- TP (True Positive): Instances that are actually positive and correctly predicted as positive.
- TN (True Negative): Instances that are actually negative and correctly predicted as negative.
- FP (False Positive): Instances that are actually negative but incorrectly predicted as positive.
- FN (False Negative): Instances that are actually positive but incorrectly predicted as negative.

## 3. Results and Discussion

**Figure 2:**
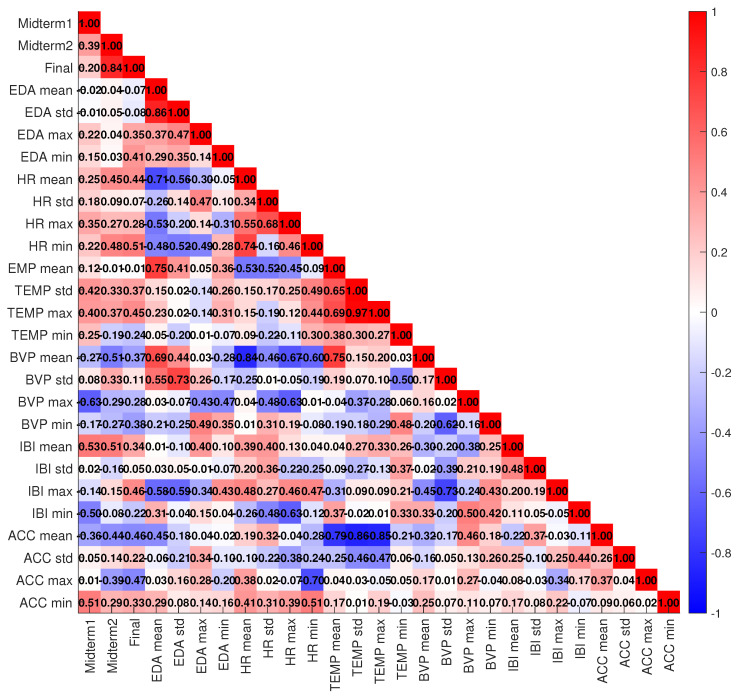
Correlation between stress indicators and exam scores. The correlation coefficients are color-coded: shades of red represent positive correlations, while shades of blue represent negative correlations. Darker shades indicate stronger correlations.

This section presents and discusses the performance of various machine learning models, consisting of Random Forest, SVM, XGBoost, CatBoost, and GBM used to predict stress observing in physiological signals affecting academic performance. As a complementary analysis and before evaluating the models’ performances, we conducted a correlation analysis to examine relationships between the estimated features as stress indicators and academic performance. This helps identify relevant features for inclusion in the model, enhancing predictive power and reducing noise. The coefficients of correlation are color-coded in Figure 2 such that the red shade shows the positive correlation, whereas the blue shade shows the negative correlation. The darker the shade stronger the correlation. It should be noted that the correlation coefficients have been estimated before applying PCA. According to Figure 2, the performance in all exam sessions positively correlated with all heart rate features. Specifically, a strong positive correlation was observed between the minimum HR, as an indication of a more relaxed state, and the performances of the midterm 2 and final exams. A significant increase in the correlation between the minimum HR and performance in the final exam, which is the longest and most important exam, reveals that well-prepared students, experiencing lower stress, tend to exhibit a lower minimum HR. Additionally, performance in all exam sessions was positively correlated with the inter-beat interval mean. Such a state can reflect optimal vigilance, where the student is focused but not overly stressed, thus supporting better cognitive performance. Taken together, these observations confirm the conclusion drawn above that higher heart rate variability and longer inter-beat intervals are strongly linked with better performance. The correlation between the maximum, mean, and standard deviation of skin temperature and performance in all exams was also positive. Specifically, the maximum temperature of well-prepared students was significantly linked with performance in the final exam. This may be a sign of a lower stress level or higher emotional regulation during the exam. Like HRV, a higher skin temperature is commonly linked to a more relaxed but alert physiological state. Their higher skin temperature may also reflect lower stress and more efficient thermoregulation. In contrast, performance in midterm 2 and the final exam was negatively correlated with minimum skin temperature. Additionally, we observed negative correlations between the mean, maximum, and minimum values of blood volume pulse and performance across all exams. The results also indicated a negative correlation between the minimum inter-beat interval and exam scores. A decrease in skin temperature, an increase in skin conductance (i.e., EDA) due to sweating, variability of blood volume pulse, and a decrease in inter-beat interval are all physiological signs of heightened stress, which may contribute to lower performance. On the other hand, students who are less prepared may experience elevated stress levels, which manifest as a decreased inter-beat interval and reduced blood volume pulse due to increased heart rate and vasoconstriction. Additionally, a negative correlation between the mean of ACC and performance in all exam sessions was observed. This may have resulted from increased body movements, which were captured as higher accelerometer activity, due to heightened stress in the students. The measure correlations also indicated a positive correlation between the minimum of measured acceleration and exam scores suggesting that students who showed lower minimum movement (i.e., periods of stillness/relaxation) tended to perform better. This could mean that reduced restlessness (low stress, calm state) during the exam period is associated with better cognitive performance. On the contrary, a negative correlation of maximum measured acceleration with exam scores means that students who showed higher maximum movement (e.g. stress-induced movements) tended to perform worse. This aligns with the idea that physical restlessness may reflect higher stress or distraction, which negatively impacts performance.

After exploring the correlations among the physiological predictors and exam scores, the performance of the models in predicting the label of each exam score was evaluated. Table 4 represents the models’ performances obtained by averaging the performance indexes across the folds.

**Figure 3:**
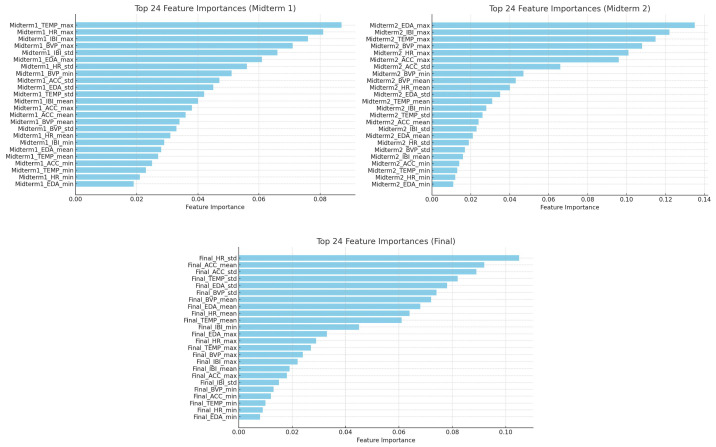
SHAP summary plot for each exam session. The plot illustrates the feature importance and impact on the model’s output. Features are ranked from most to least important on the y-axis, and the distribution of their impact on the model’s prediction is shown on the x-axis.

According to the obtained results, Random Forest performed moderately with basic hyperparameter tuning, achieving a maximum accuracy of 76% for Midterm 2. However, incorporating ANOVA feature selection and dimensionality reduction significantly improved results for midterm 1, where accuracy increased from 65% to 91%, and the F1 score rose from 0.56 to 0.91. This indicates that Random Forest benefits strongly from preprocessing techniques that reduce feature noise. SVM showed robust performance when combined with SMOTE, reaching 92% accuracy on Midterm 2. The recall, precision and F1 score were similarly high, suggesting that class imbalance correction is effective for this model. Interestingly, while the combination of ANOVA and dimension reduction yielded perfect classification on midterm 1 (100% across all metrics), its performance deteriorated on midterm 2 and the final exam, showing lower generalizability in imbalanced or complex datasets. XGBoost, using default hyperparameters, delivered balanced but modest performance (around 63% accuracy on the midterms). The application of SMOTE drastically improved midterm 1 results (accuracy jumped to 92%), but performance declined or remained unchanged in the other exams, suggesting a sensitivity to noise introduced by oversampling. CatBoost exhibited strong improvements with SMOTE across all exam sessions. Accuracy increased from 73% to 100% on midterm 1 and from 71% to 95% on midterm 2, and from 67% to 78% in the final exam, along with consistent gains in precision and F1 score. Without SMOTE, performance was inconsistent, especially for more complex datasets, highlighting the importance of addressing class imbalance in gradient boosting models. GBM showed the most improvement when combined with ANOVA and dimensionality reduction. Midterm 1 accuracy increased from 65% to 93%, and F1 score improved from 0.51 to 0.93. These results demonstrate that GBM, like Random Forest, benefits from the elimination of redundant or irrelevant features. Gains were also observed in midterm 2 and the final exam, though they were more modest, reflecting dataset-specific limitations. In summary, the results indicate that preprocessing via ANOVA and dimensionality reduction is highly effective for tree-based models like Random Forest and GBM. SMOTE yields significant performance boosts for SVM and CatBoost. XGBoost is less stable with SMOTE and may require more careful noise handling. The key distinction between CatBoost and XGBoost is their treatment of categorical variables. XGBoost does not natively support categorical features, requiring preprocessing techniques such as one-hot or label encoding, which can increase dimensionality and sometimes decrease performance. In contrast, CatBoost incorporates categorical features directly through ordered target statistics, a strategy that mitigates target leakage and often yields superior predictive accuracy when categorical attributes are important. This approach avoids the common issue of target leakage or prediction shift that can occur when using a technique like SMOTE. This may be one of the most important reasons of outperforming Catboost compared to XGboost. The best overall result was achieved for Midterm 1 by CatBoost when using SMOTE, and by SVM when using ANOVA and dimensionality reduction. CatBoost with SMOTE consistently outperformed other models across all exams. It is the most reliable and generalizable model in our analysis. XGBoost and SVM exhibited signs of overfitting, performing well in the midterms but poorly in the final, indicating limited generalization. For CatBoost with resampling, the maximum F1-score reached 100% in midterm 1, while the maximum F1-score in [17] with dropout was 87%. On the other hand, XGBoost with dropout achieved an F1-score of 88% in [19]. In our study, when XGBoost was applied with the resampling technique, its F1-score improved to 91% in Midterm 1. GBM achieved an accuracy of 90% in [18] when stress was predicted in students. However, when GBM was incorporated with ANOVA and dimension reduction techniques, its accuracy improved in some scenarios, reaching 93% in Midterm 1. XGBoost achieved an accuracy of 84.5% in [20] when mental stress among college students was evaluated using heart rate and hand acceleration. In our study, when XGBoost was tuned and the resampling technique was applied, its accuracy improved in some scenarios, reaching 92% in midterm 1. We also conducted a SHapley Additive exPlanations (SHAP) analysis to interpret the model’s decisions. This allowed us to visualize the contribution of each feature and understand how they collectively influenced the model’s predictions. Figure 3 shows the most important features that have a major impact on the model’s output. According to the SHAP output, the features for midterm 1 ranked by influence are: maximum skin temperature, heart rate, inter-beat interval, and blood volume pulse. For midterm 2, the ranked features are: amplitude of electrodermal activity, inter-beat interval, skin temperature, blood volume pulse, heart rate, and accelerometer readings. For the final exam, the features ranked by influence are heart rate variation, mean acceleration, acceleration, skin temperature, and electrodermal activity variations.

While this study provides valuable insights into the association between stress indicators measured through physiological signals and student academic performance, some limitations should be acknowledged. First, the sample size was relatively small and included only 10 participants. Additionally, the dataset includes two females and eight males which lead to gender imbalance. The second limitation concerns the weaker performance of the machine learning models in predicting exam scores in midterm 2 and the final session compared to midterm 1. The deterioration in results observed for midterm 2 and the final exam is likely due to the increased vulnerability of physiological signals to noise and artifacts under exam conditions. As the same preprocessing pipeline was applied across all sessions, its limited effectiveness in removing such noise may have disproportionately affected these sessions. This highlights the need for more advanced noise reduction methods, which we plan to pursue in future work. Additionally, the final exam is longer and more complicated compared to midterm 1, which makes it more challenging for the machine learning algorithms.

## 4. Conclusions

This study proposed a data processing pipeline to predict the students’ exam grades over three exam sessions. Physiological data, including heart rate, skin temperature, electrodermal activity, blood volume pulse, inter-beat interval, and accelerometer were collected using a wearable sensor during the exams. After some preprocessing, statistical features were extracted from the collected data. Then, resampling, ANOVA and dimension reduction techniques were applied to address data imbalance and remove irrelevant samples. In our study, five machine learning models were trained using the estimated features and their performance was evaluated using threefold stratified cross-validation. The obtained results indicated robust performance of CatBoost in predicting good exam grades versus bad exam grades. Building on the promising performance of CatBoost in predicting exam outcomes, this study opens several avenues for future research, such as increasing the sample size by employing more students or increasing the exam sessions. Expanding the dataset across institutions and incorporating broader stress indicators could enhance generalizability. We also plan to investigate the application of more advanced signal processing techniques to enhance signal segmentation and noise reduction. Future work may also benefit from exploring deep learning models, advanced resampling methods, ensemble techniques, and automated hyperparameter tuning to further improve prediction accuracy and robustness. In terms of practical applications, the proposed model could be integrated into a real-time dashboard or pre-exam alert system to help students and educators monitor stress and performance indicators. Moreover, the model’s predictions could assist counselors in identifying students who might be in need of specific interventions or support strategies. 

## Figures and Tables

**Table 1:** Optimal hyperparameters for each model obtained by the GridSearchCV at the first training approach.

Model	Exam	n_estimators	Max_Depth	Learning_Rate	C/reg	Kernel/Method
Random Forest	Midterm 1	100	None	–	–	–
	Midterm 2	100	None	–	–	–
	Final	100	None	–	–	–
SVM	Midterm 1	–	–	–	10	poly
	Midterm 2	–	–	–	0.1	linear
	Final	–	–	–	10	sigmoid
XGBoost	Midterm 1	100	3	0.01	–	–
	Midterm 2	100	3	0.01	–	–
	Final	100	3	0.01	–	–
CatBoost	Midterm 1	100	3	0.1	L2 = 1	–
	Midterm 2	100	3	0.01	L2 = 1	–
	Final	300	3	0.2	L2 = 5	–
GBM	Midterm 1	100	3	0.01	–	–
	Midterm 2	200	4	0.1	–	–
	Final	200	3	0.1	–	–

**Table 2:** Optimal hyperparameters for each model obtained by the GridSearchCV at the second training approach in which resampling methods were used.

Model	Exam	n_estimators	Max_Depth	Learning_Rate	C/reg	Kernel/Method
Random Forest	Midterm 1	100	None	–	-	-
	Midterm 2	100	None	–	-	-
	Final	100	None	–	-	-
SVM	Midterm 1	–	–	–	10	poly
	Midterm 2	–	–	–	0.1	linear
	Final	–	–	–	10	sigmoid
XGBoost	Midterm 1	100	3	0.01	–	–
	Midterm 2	100	3	0.01	–	–
	Final	100	3	0.01	–	–
CatBoost	Midterm 1	100	7	0.2	L2 = 3	–
	Midterm 2	100	3	0.2	L2 = 3	–
	Final	300	5	0.2	L2 = 3	–
GBM	Midterm 1	100	3	-	–	–
	Midterm 2	200	3	-	–	–
	Final	200	3	-	–	–

**Table 3:** Optimal hyperparameters for each model obtained by the GridSearchCV at the third training approach in which resampling and dimension reduction methods were used.

Model	Exam	n_estimators	Max_Depth	Learning_Rate	C/reg	Kernel/Method
Random Forest	Midterm 1	100	None	–	–	–
	Midterm 2	100	None	–	–	–
	Final	100	None	–	–	–
SVM	Midterm 1	–	–	-	0.01	linear
	Midterm 2	–	–	–	0.01	rbf
	Final	–	–	–	0.01	poly
XGBoost	Midterm 1	100	3	0.01	–	–
	Midterm 2	100	3	0.01	–	–
	Final	100	3	0.01	–	–
CatBoost	Midterm 1	100	3	0.2	L2 = 3	–
	Midterm 2	100	3	0.2	L2 = 3	–
	Final	300	5	0.2	L2 = 3	–
GBM	Midterm 1	100	3	-	–	–
	Midterm 2	200	3	-	–	–
	Final	200	3	-	–	–

**Table 4:** Measured evaluation metrics across models, training strategies and exam sessions.

Model	Technique	Metric	Midterm 1	Midterm 2	Final
Random Forest	Hyperparameters tuning	Accuracy	0.65	0.76	0.59
		F1 Score	0.56	0.66	0.42
		Precision	0.56	0.58	0.36
		Recall	0.65	0.76	0.59
	SMOTE	Accuracy	0.47	0.62	0.51
		F1 Score	0.46	0.51	0.38
		Precision	0.49	0.48	0.33
		Recall	0.47	0.62	0.51
	ANOVA + Dimension reduction	Accuracy	0.91	0.69	0.61
		F1 Score	0.91	0.65	0.59
		Precision	0.94	0.62	0.58
		Recall	0.91	0.70	0.61
SVM	Hyperparameters tuning	Accuracy	0.66	0.65	0.57
		F1 Score	0.51	0.51	0.40
		Precision	0.43	0.42	0.38
		Recall	0.66	0.65	0.60
	SMOTE	Accuracy	0.75	0.92	0.69
		F1 Score	0.69	0.91	0.72
		Precision	0.69	0.94	0.83
		Recall	0.75	0.92	0.70
	ANOVA + Dimension reduction	Accuracy	1.00	0.58	0.55
		F1 Score	1.00	0.52	0.42
		Precision	1.00	0.52	0.35
		Recall	1.00	0.58	0.58
XGBoost	Hyperparameters tuning	Accuracy	0.63	0.62	0.59
		F1 Score	0.50	0.48	0.52
		Precision	0.41	0.41	0.46
		Recall	0.64	0.62	0.59
	SMOTE	Accuracy	0.92	0.50	0.52
		F1 Score	0.91	0.53	0.55
		Precision	0.94	0.45	0.46
		Recall	0.92	0.55	0.59
	ANOVA + Dimension reduction	Accuracy	0.90	0.61	0.62
		F1 Score	0.90	0.62	0.61
		Precision	0.94	0.59	0.60
		Recall	0.90	0.65	0.62
CatBoost	Hyperparameters tuning	Accuracy	0.73	0.71	0.67
		F1 Score	0.68	0.59	0.54
		Precision	0.74	0.56	0.57
		Recall	0.73	0.64	0.62
	SMOTE	Accuracy	1.00	0.95	0.78
		F1 Score	1.00	0.94	0.78
		Precision	1.00	0.98	0.89
		Recall	1.00	0.96	0.78
	ANOVA + Dimension reduction	Accuracy	0.94	0.79	0.72
		F1 Score	0.94	0.72	0.71
		Precision	0.97	0.73	0.70
		Recall	0.93	0.79	0.72
GBM	Hyperparameters tuning	Accuracy	0.65	0.75	0.70
		F1 Score	0.51	0.66	0.59
		Precision	0.42	0.59	0.50
		Recall	0.65	0.77	0.70
	SMOTE	Accuracy	0.54	0.70	0.62
		F1 Score	0.52	0.64	0.54
		Precision	0.54	0.69	0.57
		Recall	0.54	0.69	0.62
	ANOVA + Dimension reduction	Accuracy	0.93	0.78	0.75
		F1 Score	0.93	0.71	0.71
		Precision	0.97	0.72	0.72
		Recall	0.92	0.77	0.72

## Data Availability

The raw data is publicly available in Physionet website: https://physionet.org/content/wearable-exam-stress/1.0.0/ (accessed on 7 September 2025).
